# Encoding of interdependent features of head direction and angular head velocity in navigation

**DOI:** 10.1093/pnasnexus/pgaf320

**Published:** 2025-10-09

**Authors:** Dongqin Cai, Tao Liu, Jia Liu

**Affiliations:** Tsinghua Laboratory of Brain and Intelligence, Department of Psychological and Cognitive Sciences, 9th Floor, Lv Dalong Building, Tsinghua University, Haidian District, Beijing 100084, China; Tsinghua Laboratory of Brain and Intelligence, Department of Psychological and Cognitive Sciences, 9th Floor, Lv Dalong Building, Tsinghua University, Haidian District, Beijing 100084, China; Tsinghua Laboratory of Brain and Intelligence, Department of Psychological and Cognitive Sciences, 9th Floor, Lv Dalong Building, Tsinghua University, Haidian District, Beijing 100084, China

**Keywords:** head direction, angular head velocity, monophasic and multiphasic tuning, dense and sparse coding, mixed selectivity

## Abstract

To navigate a dynamically changing environment, the brain must encode not only individual sensory or motor features but also their temporal interdependencies. A canonical example of this encoding challenge is the head direction (HD) system, where the current HD must be continuously updated based on its temporal derivative, angular head velocity (AHV). While previous studies have predominantly addressed the encoding of independent features, the neural representation of interdependent features remains inadequately characterized. Here, we investigated the population coding strategy for HD and AHV through a two-stage approach. First, a recurrent neural network was employed as a hypothesis-generating tool, trained to predict HD from AHV inputs, thus enabling analysis of the emergent neural responses. We observed two functionally distinct subpopulations: single-peaked (SP) units that precisely encode HD, and multipeaked (MP) units that preferentially represent AHV. Notably, both subpopulations exhibited mixed selectivity, yet diverged in their functional specialization. Then, empirical validation using neurophysiological recordings from the mouse HD system confirmed that SP and MP neurons indeed differentially encode HD and AHV. Neural geometric analysis further revealed that MP neurons expanded the dimensionality of the neural representation space, enabling a higher-resolution encoding of AHV. This configuration effectively balances the conflicting demands of specificity, achieved via sparsity, and interdependency, realized via mixed selectivity, forming a hybrid coding scheme previously uncharacterized for interdependent features. These findings together propose a hybrid coding scheme in the mouse HD system for representing dynamically coupled features and advocate future exploration of this scheme across sensorimotor and cognitive domains.

Significance Statement Neural systems often face the challenge of encoding features that are mathematically and behaviorally interdependent. A prominent example is the head direction (HD) system, where the brain must simultaneously represent HD and its temporal derivative, angular head velocity (AHV). Using computational modeling alongside empirical mouse neurophysiological recordings, we show that neurons exhibiting mixed selectivity self-organize into two functionally distinct subpopulations: one optimized for stable HD encoding and the other for dynamic AHV integration. This hybrid code effectively expands the representation dimension, enabling high-resolution AHV encoding while preserving directional specificity. Collectively, our study reveals a hybrid coding scheme for representing interdependent features in the mouse HD system, providing a candidate framework for investigating similar mechanisms across sensorimotor and cognitive domains.

## Introduction

Consider navigating a winding path while riding a bicycle. To maintain a stable sense of direction, the brain must track not only the current head direction (HD) but also the rate at which this direction changes, that is, the temporal derivative of HD, angular head velocity (AHV). This exemplifies interdependent features, where the instantaneous value of one variable (HD) is directly influenced by the other (AHV). Simultaneous encoding of such variables presents a fundamental computational challenge: the system must preserve the specificity of individual feature while maintaining their interdependency, a balance crucial for accurate prediction, integration, and control.

Neurons encoding HD and AHV are widely distributed across numerous cortical and subcortical regions in mammals. Specifically, HD neurons are predominantly located within the limbic (1–6) and extralimbic areas (7–10), whereas AHV neurons are identified in peripheral nuclei such as the lateral mammillary nucleus (3) and dorsal tegmental nucleus (11). Recent studies reveal that AHV neurons coexist with HD neurons in motor (10), visual (12), and retrosplenial cortices (8), suggesting brain-wide conjunctive encoding of HD and AHV (4, 7, 8, 13).

**Fig. 1.**
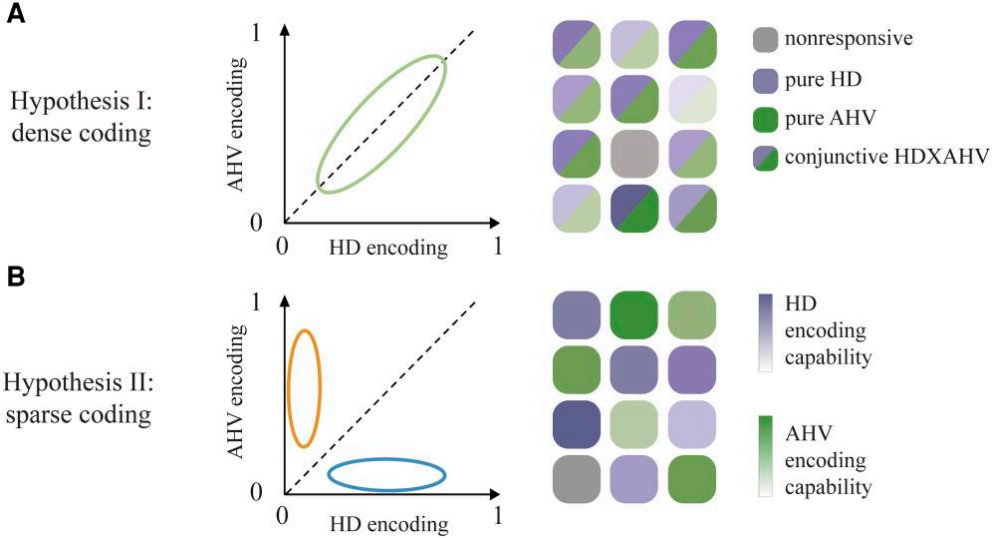
Two hypothetic coding schemes for simultaneously encoding HD and AHV. A) Dense coding. HD and AHV are represented by a homogeneous neuronal population exhibiting mixed sensitivity, with neurons showing varying sensitivities to both features. The interdependency between HD and AHV is evident at the single-neuron level through mixed response profiles and at the population level through distinct activation patterns. B) Sparse coding. HD and AHV are encoded by two functionally specialized neuronal populations, each selectively tuned either to HD or AHV. The interdependency emerges primarily at the population level, as the coordinated activity of the distinct neuron populations integrates the two features. In both panels, each square depicts the response profile of an individual neuron, with darker colors indicating stronger tuning strength or greater information content and lighter colors indicating weaker tuning or lower content.

Previous studies have predominantly investigated independent sensory or motor features, such as shape and color in vision, or frequency and intensity in auditory processing, which can be encoded orthogonally and decoded independently (14, 15). In these contexts, two canonical neural coding schemes have been widely discussed: dense coding, where individual neurons exhibit mixed selectivity and respond to multiple features simultaneously, providing flexibility and efficiency (16–20); and sparse coding, where neurons exhibit sharply defined selectivity for individual feature, enhancing precision and separability (21, 22). Here, we examined whether these two coding schemes extend to the encoding of interdependent variables such as HD and AHV (Fig. 1). Specifically, dense coding would suggest that neurons simultaneously encode both HD and AHV, reflecting variable interdependency at the single-neuron level (Fig. 1A). Sparse coding, on the other hand, would propose distinct neuronal populations that specialize separately in encoding either HD or AHV, with their interdependency emerging at the population level through coordinated activity (Fig. 1B).

To empirically investigate these two coding schemes, we first used computational modeling approach to systematically explore emergent solutions to simultaneously encode HD and AHV, and then validated these solutions through analysis of neural recordings from the mouse HD system. Specifically, we employed a recurrent neural network (RNN) explicitly trained to simultaneously encode HD and AHV. This RNN, inspired by similar models in previous studies examining conjunctive encoding of task-related variables (23, 24), allowed us to isolate the self-organizing principles underlying HD and AHV representation, mitigating potential confounding factors from interactive influences of upstream and downstream cortical regions or from intrinsic functionalities not directly related to HD and AHV within biological HD systems. This modeling identified two distinct subpopulations of units with single-peaked (SP) and multipeaked (MP) tuning curves, specialized respectively in HD and AHV encoding, suggesting a hybrid coding scheme that combines the representational flexibility of mixed selectivity with a functional division of labor at the population level. Subsequent analysis of neurophysiological recordings from mouse cortical and subcortical HD regions validated this hybrid coding framework, revealing a similar functional division of labor between SP and MP neurons. Finally, neural geometric analysis indicated MP neurons effectively increased the dimensionality of the neural representational space, thereby facilitating high-precision representations of AHV.

## Results

### Emergence of SP and MP units for HD in RNN of HD system

**Fig. 2:**
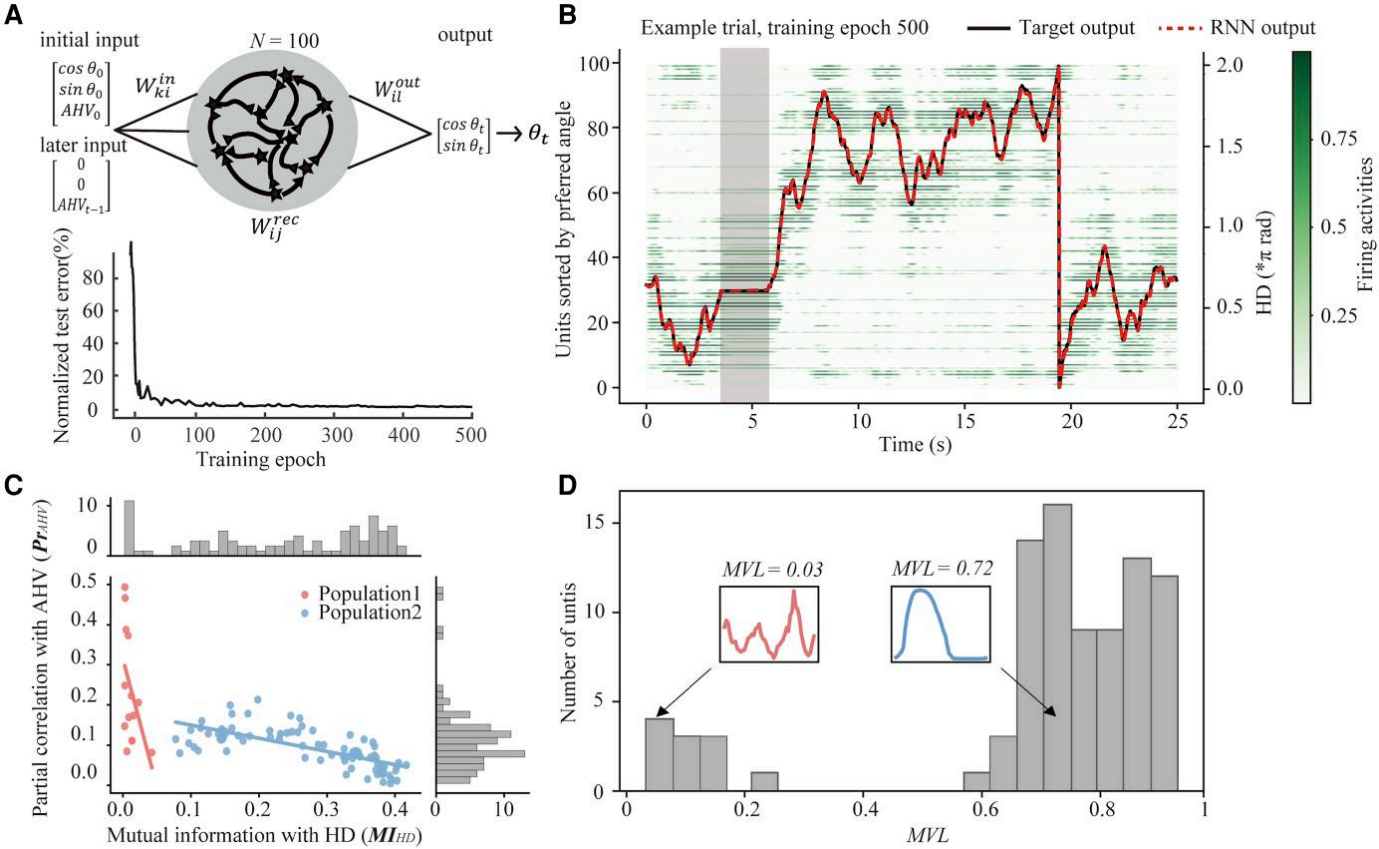
RNN model and the emergence of SP and MP units. A) Top: Schematic illustration of the RNN, trained to predict real-time HD $\theta_{t}$ from initial HD and real-time AHV inputs. Bottom: Normalized mean-squared errors on testing trials across training epochs, shown relative to the initial epoch. B) Example test trial demonstrating superimposed traces of RNN-predicted HD, ground truth HD, and normalized firing rates of individual units. The shaded region shows stable HD prediction when AHV is set to zero. C) Scatter plot depicting the relationship between HD encoding (mutual information, MI_HD_) and AHV encoding (absolute partial Pearson correlation coefficient, Pr_AHV_) for individual units. Two distinct populations emerge: Population 1 (linear regression slope *ρ* = −6.35, *P* = 0.08) consists of 11 MP units and 2 SP units with exceptionally low firing rates (0.0087 and 0.025), whereas Population 2 (linear regression slope *ρ* = −0.33, *P* < 0.05) comprises exclusively 75 SP units. Marginal distributions (histograms) show the distribution of MI_HD_ (top) and Pr_AHV_ (right). D) Histogram showing MVL distribution for all HD-tuned units. Insets display exemplar SP and MP tuning curves with corresponding MVL values, highlighting the bimodal distribution that distinguishes the two populations.

We adopted an RNN (Fig. 2A, top), which was used to model rodent and fruit fly HD systems (23), to uncover the self-emergent solutions that allow for the simultaneous encoding of HD and AHV. In addition to its recurrent architecture that simulates neuronal connectivity, the dynamics of each unit in the RNN were regulated by the firing rate model (25) to simulate the firing characteristics of neurons. Inputs to the model consisted of three components: two inputs encoding the initial HD in the form of $\sin\;\theta_{0},\cos\;\theta_{0}$, and one scalar input representing either clockwise (negative) or counterclockwise (positive) AHV at each timestep. The output of the model was the predicted HD based on these inputs. The model was trained using the Hessian-free algorithm (26) and optimized through stochastic gradient descent to minimize the mean-squared error between RNN-predicted HD values and target HD values derived directly through AHV integration.

During each training epoch, AHV inputs were from simulated datasets whose distributions matched those empirically observed in rodents (3, 27). Training was conducted on 500 trials, each consisting of 500 timesteps, with performance assessed on an additional set of 500 unseen trials of equal length. Test-trial prediction errors decreased drastically within the first 10 epochs and reached near-minimal levels by approximately epoch 300 (Fig. 2A, bottom). After extensive training for 500 epochs, the RNN showed robust tracking of HD changes in response to varying AHV inputs (Fig. 2B) and maintained stable HD representations when AHV was set to 0 (Fig. 2B, the shaded region). Consequently, we used this trained model to generate a simulated session consisting of 2,00,000 timestep (∼80 min of practical recording) for subsequent analyses.

We first examined how HD and AHV were encoded at the single-unit and population levels, taking advantage of complete unit activity data afforded by computational modeling. The encoding strength for HD was quantified by calculating the mutual information (MI, bits/s) between HD and each unit's averaged firing rate (28), referred to as MI_HD_. This metric measures shared entropy between two dependent variables (7, 29), with higher values indicating greater information transmission over time. Meanwhile, a unit's capacity of encoding AHV was assessed by Pr_AHV_, which is the absolute partial Pearson correlation coefficients (30) between AHV and firing rates (8, 9), computed separately for positive and negative velocities, with a higher value indicating better AHV representation. We found that a significant proportion of RNN units (84%) exhibited sensitivity to both HD and AHV to varying degrees (Fig. 2C), mirroring empirical observations of heterogenous encoding capabilities (7, 23). Specifically, we observed a significant negative correlation between MI_HD_ and Pr_AHV_ across the entire population (*r* = −0.67, *P*  *<* 0.001), suggesting that units adept at encoding HD tended to exhibit diminished AHV encoding capability. This finding was also replicated using a Gaussian generalized linear model (31) with sine and cosine predictors for HD alongside AHV as covariates (Fig. S1A and B).

Upon closer examination, we found that the decline in Pr_AHV_ was pronounced steeper for units with lower MI_HD_ (population 1: *R*² = 0.24, *P* = 0.08) compared to those with higher MI_HD_ (population 2: *R*² = 0.49, and *P* < 0.001; Fig. 2C). This relationship was best captured by a two-stage piecewise linear function, with a steeper fitted slope (*ρ* = −16.9) for MI_HD_ values below 0.025, and a gentler slope (*ρ* = −0.28) for MI_HD_ values above 0.025. This two-stage piecewise linear function (*R*^2^ = 0.60) outperformed single-stage linear (*R*^2^ = 0.50), quadratic (*R*^2^ = 0.48), or exponential (*R*^2^ = 0.49) models. Additionally, the robustness of this distinction was also confirmed using MI normalized by mean firing rate (bits/spike; Fig. S1C), supporting the presence of two functionally distinct neuronal populations of divergent conjunctive coding patterns for HD and AHV.

Given the known influence of tuning curve shape on neuronal encoding properties (32, 33), we subsequently examined whether the two distinct populations demonstrated unique tuning characteristics. To do this, HD tuning curves were constructed from firing rates across all timesteps (Fig. S2A and B). We found that units with higher MI_HD_ predominantly exhibited monophasic tuning curves, characterized by sharply peaked HD selectivity (Fig. S2A, blue curves), hence labeled as SP units. In contrast, units with lower MI_HD_ exhibited multiphasic tuning curves, displaying multiple distinct response peaks across different HDs (Fig. S2A, red curves), hence labeled as MP units. To quantify this divergence, we calculated the mean vector length (MVL or Rayleigh *r*) for each unit (9, 34), where higher MVL value signified monophasic selectivity and lower values reflected multiphasic responses. The MVL distribution showed a clear bimodal pattern (Fig. 2D), with monophasic tuning curves predominating (87.5% of units, MVL > 0.5). In contrast, units with multiphasic tuning curves (MVL < 0.5) constituted ∼10% of the population. Notably, MP units were functionally critical to network performance, as their selective deactivation resulted in significantly larger increase in HD prediction error than deactivation of an equivalent number of SP units (Fig. S3). This structural dichotomy persisted even upon downsizing the RNN model (Fig. S2D and E), highlighting its robustness.

To further quantify encoding capabilities of SP and MP units, we employed a deep feedforward artificial neural network (ANN) (for details, see Materials and methods), a decoding approach well-suited for exacting maximal information from neuronal firing patterns (35–37). This approach has demonstrated high reliability, with strong generalization from training to testing datasets and minimal variance across different cross-validation splits and simulation sessions (37, 38). Separate ANNs were trained to decode either HD or AHV values from SP or MP population activities within simulated RNN sessions. Pearson's correlation coefficient between ANN-predicted and actual HD (*r*_HD_) or AHV (*r*_AHV_) values served as a metric for population-level encoding capability.

**Fig. 3.**
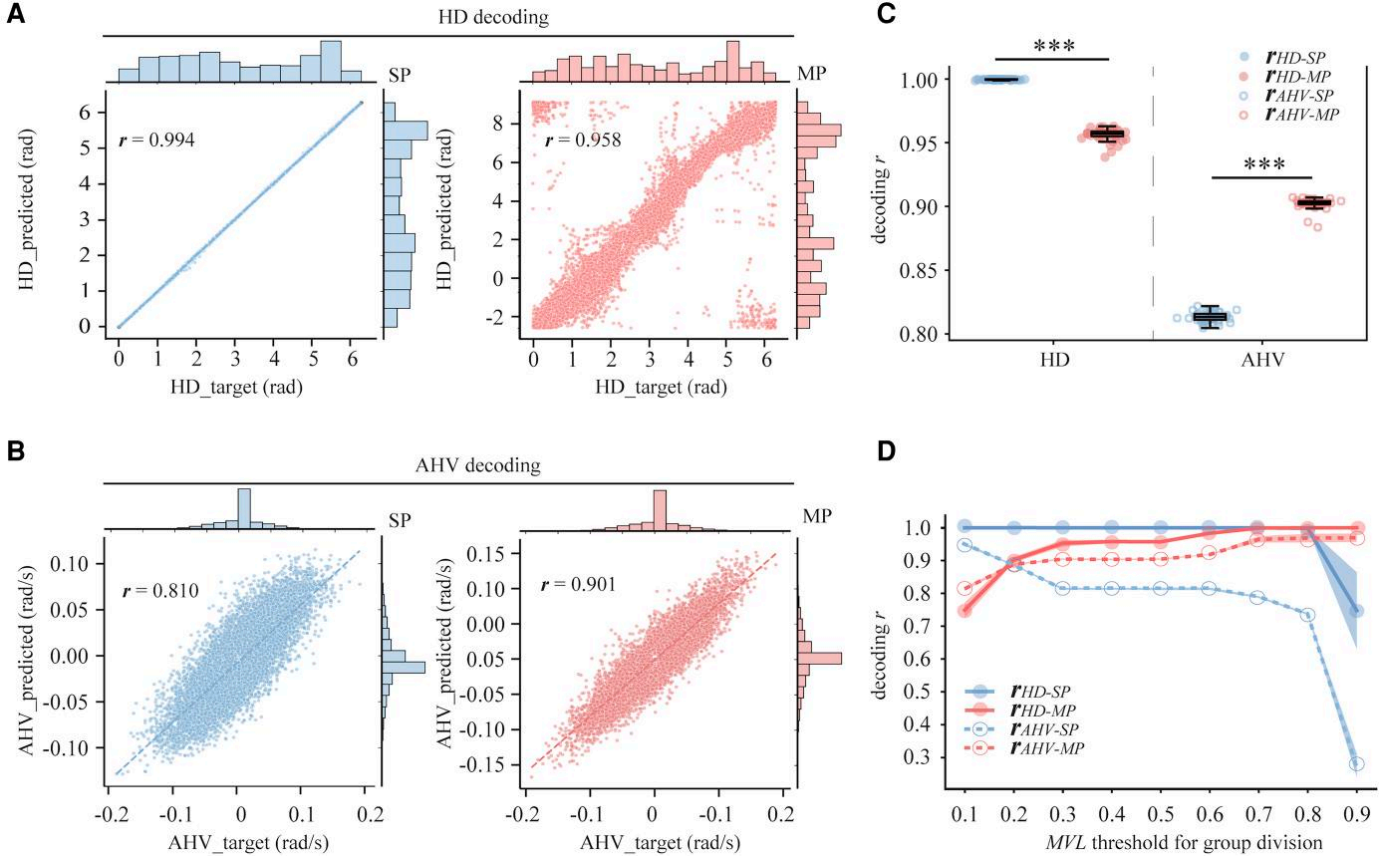
Dissociation of SP and MP populations in HD and AHV encoding. A, B) Scatter plots illustrating correlations between ANN-predicted and actual values for HD (A) and AHV (B) from a representative simulated session. Pearson correlation coefficients (*r*) indicate decoding accuracy. Dashed lines denote linear fits. Marginal histograms denote distributions of predicted and actual values. C) Scatter and box plots summarizing decoding *r* values across 50 simulated sessions with varying random seeds, comparing SP vs. MP populations for HD and AHV. A significant interaction highlights functional specialization that SP units demonstrate superior HD encoding, and MP units excel in AHV encoding. Error bars denote SD. ***P  *<* 0.001, Mann–Whitney *U* test. D) Decoding *r* values as a function of varying MVL thresholds used to categorize units into SP (MVL above threshold) and MP (MVL below threshold) populations. Solid and dashed lines represent mean decoding *r* values for HD and AHV, respectively, across 20 simulated sessions. Shaded areas represent SD. All comparisons were statistically significant (*P* < 0.05, Mann–Whitney *U* test with Holm–Bonferroni correction), except for AHV encoding between SP and MP at an MVL threshold of 0.2.

Figure 3A and B shows the correlation between predicted and actual data from a representative simulated session, highlighting the distinct encoding capacities of SP and MP unit populations for HD and AHV, respectively. Specifically, the SP population demonstrated superior HD encoding than the MP population (decoding *r*_HD_: 0.99 vs. 0.96), consistent with previous studies associating lower decoding accuracy with neurons characterized by more uniformly distributed tuning curves (37). In contrast, the MP unit population exhibited enhanced AHV encoding capacity (decoding *r*_AHV_: 0.90 vs. 0.81). This dissociation in encoding strength was further illustrated by decoding accuracy distributions derived from 50 simulated sessions initialized with different random seeds (Fig. 3C). These distributions showed opposite patterns of encoding performance for HD and AHV between SP and MP populations (decoding *r*_HD_: SP = 1.00 vs. MP = 0.96, *P*  *<* 0.001; decoding *r*_AHV_: SP = 0.81 vs. MP = 0.90, *P*  *<* 0.001; Mann–Whitney *U* test). A significant two-way interaction of unit type (SP vs. MP) by encoded variable (HD vs. AHV) further confirmed these findings [F(1, 48) = 17572, *P*  *<* 0.001]. Moreover, classifying units into SP and MP populations based on varying MVL thresholds revealed that even a small cohort of MP units with the lowest MVL values outperformed the remainder with higher MVL values in AHV encoding. In contrast, SP units with moderate MVL thresholds (e.g. 0.7–0.8) achieved near-perfect decoding accuracy for HD. However, SP units with extremely high MVL thresholds (e.g. 0.9), despite having sharper directional tuning, demonstrated significantly reduced decoding performance due to sparse HD coverage (Fig. 3D).

In summary, computational modeling revealed two functionally distinct populations within the RNN: SP units specialized primarily in HD encoding and MP units excelling in AHV representation. This emergent neural organization therefore offers a testable hypothesis of a hybrid coding scheme that may be employed by biological HD system to represent the dynamically coupled variables of HD and AHV.

### Dissociation between SP and MP neurons in encoding HD and AHV in the mouse HD system

**Fig. 4.**
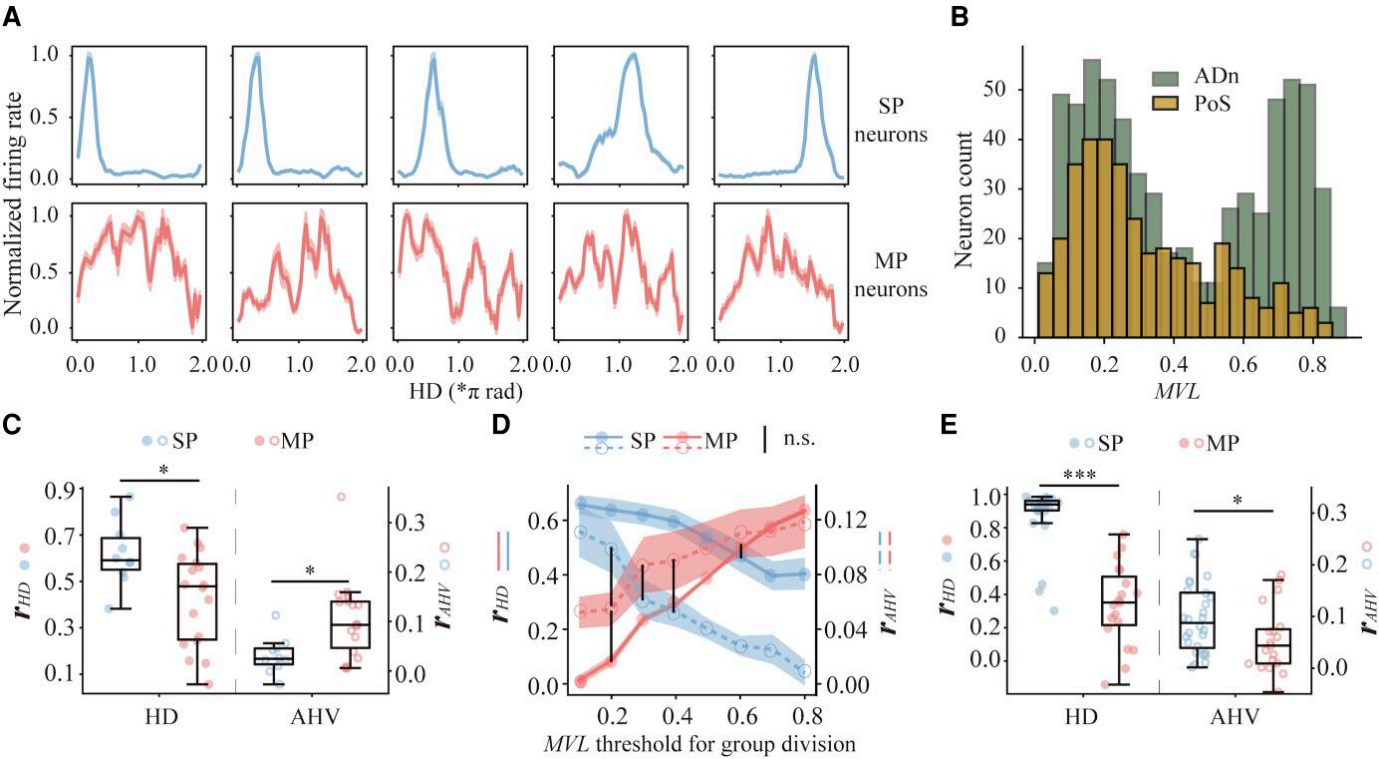
The dissociation of encoding HD and AHV between SP and MP neurons in mouse cortical and thalamic HD system. A) Tuning curves of representative neurons from a single recording session in PoS, illustrating neurons with the highest MI_HD_ values (SP neurons, top panel) and the lowest MI_HD_ values (MP neurons, bottom panel). B) Distribution of MVL values for HD-tuned neurons in ADn and PoS, revealing a clear bimodal organization in both regions, which separates putative SP (high MVL) and MP (low MVL) neuronal populations. C) Scatter and box plots comparing decoding *r* of SP and MP neurons for HD and AHV in PoS. Error bar: SD. D) Decoding *r* as a function of varying MVL thresholds for classifying PoS neuron into SP and MP populations. Solid (*r*_HD_) and dashed (*r*_AHV_) lines represent mean decoding performance for HD and AHV, respectively, across all sessions. All comparisons reached statistical significance (*P* < 0.05, Mann–Whitney *U* test with Holm–Bonferroni correction), except for those connected by vertical black lines. E) Scatter and box plots illustrating decoding *r* between SP and MP neurons in ADn for HD and AHV. *P  *<* 0.05; ***P  *<* 0.001.

Interestingly, both SP and MP neurons tuned to HD have previously been identified across several mouse cortical regions, including the anterior dorsal nucleus (ADn), Postsubiculum (PoS), retrosplenial cortex, and entorhinal cortex (7, 39). Previous studies have primarily focused on SP neurons, possibly due to their prevalence, clearly defined selectivity, and simplicity in computational modeling (40, 41). Motivated by the results obtained from our RNN model, we investigated neural representations of HD and AHV within the PoS, the primary cortical region in the mouse HD system (27, 42, 43), where neurons exhibit conjunctive sensitivity to both HD and AHV (44). Utilizing publicly available neural data from CRCNS.org (45), which includes single-unit firing activities from PoS neurons during free-foraging behavior, we found that 78% of neurons exhibited significant HD tuning, and within this subset, 64% were concurrently responsive to AHV. This proportion of neurons conjunctively encoding both HD and AHV exceeded previous reports (5, 44), likely due to our explicit inclusion of MP neurons, which were traditionally excluded from analysis. Figure 4A illustrates tuning curves of PoS neurons from an example recording session, displaying neurons with the highest (SP) and lowest (MP) MI_HD_ values, mirroring observations from the RNN simulations (Fig. S2A).

To investigate whether the hybrid coding scheme identified in the RNN model applies to biological HD systems, we first classified PoS neurons into SP or MP categories using a two-step classification approach with a strictness parameter α (for details, see Materials and methods). At α=0.1, this categorization aligned closely with visual inspection (Fig. S4A and C). Statistical analyses of sessions containing at least four neurons per category revealed a clear dissociation between SP and MP neurons in their encoding capacities for HD and AHV (Fig. 4C). A two-way repeated-measures ANOVA, with factors of neuron type (SP vs. MP) and encoded variable (HD vs. AHV), confirmed a significant interaction [F (1,154) = 33.86, *P* < 0.001], indicating that these neuron types preferentially encode different features. Post hoc comparisons confirmed that SP neurons outperformed MP neurons in decoding HD (*P* < 0.02), while MP neurons showed superior decoding of AHV (*P* < 0.02). This dissociation was robust across a range of classification thresholds (Fig. S4F).

We further examined the distributions of MVL values among all HD-tuned neurons. Consistent with the findings from the RNN model, MVL distributions exhibited a clear bimodal pattern, differentiating SP (high MVL) and MP (low MVL) neuronal populations (Fig. 4B). To systematically evaluate decoding capacities across varying MVL thresholds, we classified neurons into SP and MP populations and assessed HD and AHV decoding performance (Fig. 4D). In line with modeling results, MP neurons identified at a lower *MVL* threshold (∼0.2) demonstrated superior AHV encoding accuracy. Therefore, the mouse HD system apparently adopts a coding strategy similar to that identified computationally, with SP neurons specialized for HD representation and MP neurons excelling in AHV representation.

To explore potential physiological distinctions between SP and MP neuron types, we analyzed spike waveform properties within the PoS neuronal population. We found that MP neurons typically exhibited shorter trough-to-peak intervals (most below 0.5 ms), whereas SP neurons predominantly showed broader waveforms (>0.5 ms) (Fig. S6). Although definitive cell-type identification was not possible, this result suggests that SP and MP populations may differ in underlying cellular identity (46).

A more profound question is whether the functional division of labor observed in SP and MP neurons extends beyond PoS to other regions within the mouse HD system. To address this, we examined neuronal encoding of HD and AHV within mouse ADn, a thalamic nucleus relaying sensory information from peripheral nuclei to PoS. Employing the same procedure as for PoS neurons, we found a similar dissociation pattern in ADn, characterized by a significant two-way interaction between neuron type (SP vs. MP neurons) by encoded variable (HD vs. AHV) [F(1,45) = 59.77, *P*  *<* 0.001]. Unlike PoS, SP neurons outperformed MP neurons in both HD and AHV encoding, although the difference was significantly larger for HD (HD: 0.88 vs. 0.34, *P*  *<* 0.001; AHV: 0.10 vs. 0.06, *P* = 0.04; Mann–Whitney *U* test) (Fig. 4E). Taken together, the functional division of labor between SP and MP neurons in encoding HD and AHV might represent a general principle within the mammalian HD system, emphasizing the specific advantage conferred by MP neurons for AHV representation.

### MP units create high-resolution HD neural manifolds

Both computational modeling and empirical data revealed a consistent functional dissociation, with SP neurons primarily encoding HD and MP neurons preferentially encoding AHV. This dissociation raises an important mechanistic question regarding neural features enabling MP neurons to achieve superior AHV encoding relative to SP neurons. One prominent difference between SP and MP neurons was their tuning profiles: SP neurons exhibited selective responses around a single preferred HD, whereas MP neurons exhibit mixed selectivity, responding to multiple distinct directions. To examine how these tuning curve characteristics influence the geometry of neural population representations, we employed full width at half maximum (FWHM) as a direct and interpretable measure of tuning curve sharpness, thus providing a mechanistic link between individual tuning and the local resolution of HD manifolds. For MP neurons, which possessed multiple distinct peaks, FWHM was calculated for each peak individually, and their mean value was used to characterize their influence on the local representational geometry. As expected, MP neurons showed significantly narrower average FWHM compared to SP neurons across all analyzed datasets (RNN: 1.29 rad vs. 2.14 rad, *P*  *<* 0.001; PoS: 0.82 rad vs. 1.05 rad, *P*  *<* 0.001; ADn: 0.79 rad vs. 1.28 rad, *P*  *<* 0.001). Recent theoretical studies suggests that tuning curve width critically shapes the dimensionality of neural representation spaces (32, 47–49), thereby influencing the functionality of neural networks. Thus, we systematically investigated how tuning curve width impacts HD and AHV encoding, specifically leveraging the controlled environment of the RNN model to minimize confounding factors such as noise or sampling biases inherent in empirical data.

**Fig. 5.**
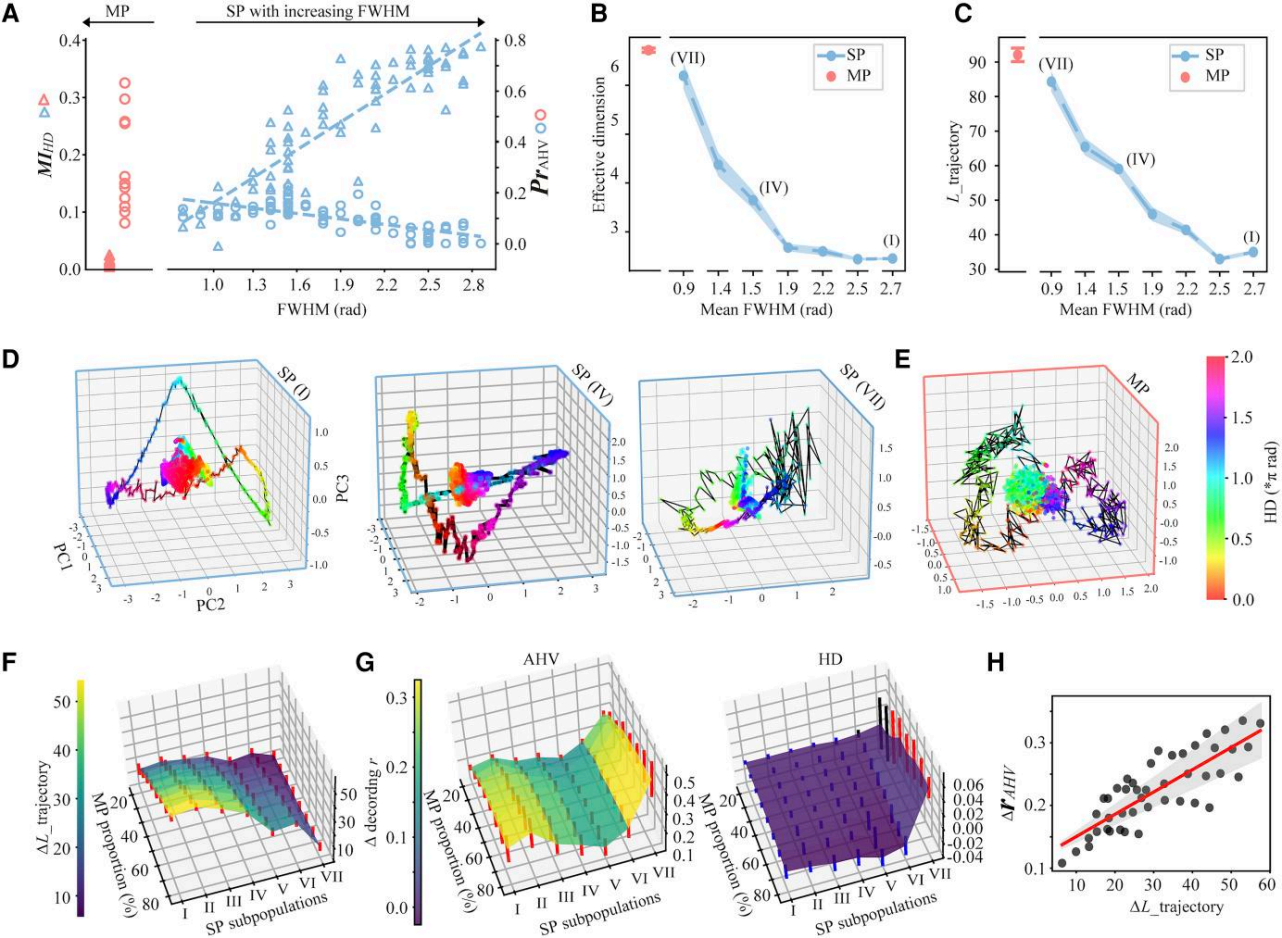
Advantages of MP units for high-resolution manifolds. A) Relationship between HD encoding (MI_HD_), AHV encoding (Pr_AHV_), and tuning curve width (FWHM) across SP and MP units in the RNN. Blue triangles represent MI_HD_ and blue circles represent Pr_AHV_ for SP units, with dotted lines indicating linear fits. Red triangles and circles represent MI_HD_ and Pr_AHV_ for MP units, respectively. Broader tuning curves in SP units correlate with higher MI_HD_ but lower AHV encoding capacity, whereas MP units display narrower tuning curves, facilitating enhanced AHV encoding. B) Effective dimensions calculated for seven SP subpopulations (ordered by increasing mean FWHM) and one MP subpopulation across 20 simulated sessions with different random seeds. FWHM for MP units is the mean width of individual peaks, allowing for direct comparison of local tuning width rather than overall angular spread. X-axis denotes mean FWHM for each SP subpopulation. C) HD manifold trajectory length ($\Delta L_{\mathrm{trajectory}}$) measured in the original 11-dimensional space for seven SP subpopulations and one MP subpopulation across 20 simulated sessions. Trajectory lengths reflect local representation resolution, with narrower-tuned SP units and MP units displaying longer trajectories indicative of higher AHV encoding resolution. D, E) Visualization of HD manifolds for SP subpopulation I, IV, and VII (as in B and C) and MP subpopulation, projected onto the first three principal components of their respective neuronal activity spaces. Colored dots connected by black lines represent mean neuronal responses across HD bins (0−2π radians). Normalized point clouds represent projected neural states from all timesteps in a simulated session. Color gradients indicate HD values as shown in the color bar in (E). F, G) Changes in manifold trajectory length ($\Delta L_{\mathrm{trajectory}}$) and decoding *r* for AHV and HD upon systematically replacing varying proportions (20–80%) of SP units with MP units, assessed for each SP subpopulation. Error bars represent SD across 20 randomized replacements. Red and blue error bars represent significant positive and negative deviations from 0, respectively, while black error bars indicate nonsignificance changes. H) Correlation between changes in HD manifold trajectory length and corresponding changes in decoding *r*_AHV_ resulting from replacing SP units with MP units for SP subpopulation I to VI, demonstrating that increases in manifold complexity consistently predict improvements in AHV representation accuracy.

First, we explored the relationship between tuning curve width and encoding capacity for HD and AHV within the SP units, intentionally excluding MP units to isolate the influence of tuning width independently from mixed selectivity. We found that tuning curve width in SP units positively correlated with MI_HD_ (*r* = 0.89, *P*  *<* 0.001), suggesting that broader tuning curves facilitate superior HD encoding (Fig. 5A, blue triangles). Notably, this observation demonstrates that increased MI does not necessarily require narrower tuning. A contributing factor is that, in both the RNN model and mouse recordings, broader SP units tend to exhibit higher firing rates as FWHM increases (Fig. S5), which can offset the effect of wider tuning on information content. In contrast, a negative correlation was observed between tuning width and Pr_AHV_ (*r* = −0.72, *P*  *<* 0.001), suggesting that narrower tuning curves improve AHV representations (Fig. 5A, blue circles). MP units, which exhibited narrower tuning width than the narrowest SP units, accordingly demonstrated enhanced AHV encoding capability (Fig. 5A, red circles).

Given the continuum of tuning widths within SP units, we further investigated the representational geometry constructed by SP unit subpopulations differentiated by their tuning widths. Specifically, SP units in the RNN were sorted according to FWHM and evenly divided into seven subpopulations, each with 11 units. Effective dimensionality (ED) (50, 51) was computed for each subpopulation's neural representation space, with higher dimensionality indicative of richer representational diversity. We found that ED monotonically increased as tuning widths narrowed [F(6, 13) = 1,485.4, *P*  *<* 0.001, Fig. 5B], implying that units with narrower tuning width contribute to high-dimensional neural representations.

To quantify difference in neural manifolds across these SP subpopulations, firing rates were z-scored to eliminate biases from response strength variability. Mean firing rates for each unit were computed across HD bins (360 bins spanning 0−2π radians), constructing HD manifolds in an 11-dimensional space (i.e. 11 units). We measured manifold trajectory lengths, a metric reflecting local representational complexity, as cumulative Euclidean distances between adjacent HD bins (for details, see Materials and methods). We found that manifold trajectory length increased significantly as tuning widths narrowed [F(6, 13) = 2138, *P*  *<* 0.001; Fig. 5C], suggesting that narrower tuning curves provide finer local resolution, thereby enabling more accurate AHV representation. Visualization of representative HD manifolds projected onto their first three principal components supported this interpretation. Figure 5D shows that exemplar HD manifolds for SP subpopulations I, IV, and VII (depicted by colored dots threaded by black lines) formed a 1-D ring representing HDs from 0 to 2π. Clearly, HD manifolds differed significantly in degree of smoothness (i.e. local structure). Specifically, narrower-tuned SP subpopulations exhibited more intricate manifold trajectories with pronounced local structure characterized by abundant twists and turns (Fig. 5D, right), indicative of higher dimensionality, whereas broader-tuned subpopulations showed smoother trajectories with reduced complexity (Fig. 5D, left).

Because AHV is the temporal derivation of HD, the rotation speed of AHV equates to the change in HDs over an extremely short period. In terms of neural geometry, the proximity between two adjacent HDs signifies AHV. Therefore, greater local structure complexity corresponds to a longer distance between two adjacent HDs, hereby better AHV representation precision. Therefore, incorporating MP units with notably narrow tuning width would represent an optimal strategy for achieving high-resolution AHV encoding. Indeed, the representational space formed by MP units exhibited the highest ED (Fig. 5B, red), the longest manifold trajectory length (Fig. 5C, red), and consequently the richest local structure complexity (Fig. 5E), thereby enabling exceptionally high-resolution AHV encoding. Further analyses demonstrated that randomly replacing 20–80% of SP units with MP units (for details, see Materials and methods) consistently increased manifold trajectory length (Fig. 5F). Importantly, increases in manifold length significantly correlated with improved AHV decoding accuracy (*r* = 0.84, *P*  *<* 0.001; Fig. 5H), whereas the corresponding reduction in HD decoding accuracy was negligible (mean change in decoding *r*: −0.008 for HD vs. 0.22 for AHV) (Fig. 5G, right).

Taken together, these results demonstrate that MP units effectively enhance the dimensionality and local representational complexity of HD neural manifolds, thereby facilitating high-resolution representations of AHV.

## Discussion

To investigate the neural mechanisms underlying the simultaneous encoding of interdependent features, HD and AHV, we developed an innovative methodological approach, with a computation model of HD systems (23) as an exploratory tool for hypothesis generation and subsequently validating emergent hypotheses with empirical neurophysiological data. Using this method, we identified two functionally distinct neuronal populations: MP neurons, which compromised HD encoding specificity to more efficiently capture AHV dynamics, and SP neurons, which predominantly encoded HD. Further neural geometric analyses revealed that tuning curve width critically influences the complexity of local neural manifolds, consequently affecting the dimensionality of the representational space and modulating representation precision according to task demands. Given the findings of conjunctive encoding of interdependent features beyond navigation, such as motor cortical neurons simultaneously representing hand position and velocity (52, 53), our study offers a candidate framework for investigating neural encoding strategies for interdependent variables across various cognitive domains.

The key factor driving the expansion of neural representational dimensionality appears to be the tuning curve width. Previous theoretical studies suggest that narrower tuning curves not only enhance the amount of information encoded by individual neurons (54), but also increase the linear dimensionality of population-level representations (32, 47) and facilitate low-latency signal transmission (33). Previously, this characteristic has primarily been investigated within the context of SP neurons, which exhibit considerable variability in tuning widths. Our findings reveal that MP neurons exhibited tuning widths similar to the narrowest SP neurons, and yet produced higher-dimensional representational space with improved encoding resolution for stimulus details (55). Thus, MP neurons appear essential for complementing SP neurons, forming a comprehensive neural coding system. Besides tuning and encoding differences, SP and MP neurons showed distinct electrophysiological signatures. Specifically, most MP neurons demonstrated shorter spike waveform trough-to-peak intervals (<0.5 ms), suggesting potential correspondence with fast-spiking interneurons. Recent empirical evidence indicates that putative inhibitory interneurons often exhibit MP tuning in spatial navigation tasks (46), a characteristic aligning with our identified MP neuronal population. Although definitive cell-type identification remains beyond the scope of our study, these results raise possibility regarding the differential cellular composition underlying these distinct functional populations.

Because HD and AHV are inherently interdependent, with AHV continuously updating the instantaneous HD, the fundamental challenge lies in balancing specificity for each individual features while simultaneously encoding their dynamic coupling. This challenge significantly differs from encoding independent sensory or motor features. To preserve the specificity of features, the HD system appears to adopt a sparse-like coding strategy, where SP neurons specialized in HD encoding and MP neurons optimized for AHV encoding. This sparse-like coding scheme may provide an efficient mechanism to maintain distinct representations, minimizing mutual interference, and ensuring the balance between interference resistance and generalization capacity (56). In addition, given that HD and AHV signals likely serve different functional purposes in downstream cortical regions, sparse-like coding offers additional advantages, such energy efficiency and simplified decoding (57, 58). In this context, SP and MP neurons resemble pure-selectivity neurons, specializing distinctly in their respective features. In contrast, to encode the interdependency between HD and AHV, the HD system also employed a dense-like coding strategy, with both SP and MP neurons demonstrating mixed-selectivity to varying degrees. In addition, although MP neurons exhibited narrowest tuning curves compared to SP neurons, SP neurons alone could encode AHV effectively, albeit less precisely than when augmented by a subset of MP neurons. In this context, SP and MP neurons differ quantitatively rather than qualitatively in their functional role, both demonstrating attributes associated with mixed selectivity. Recent studies (18–20) highlight the critical role of mixed selectivity in forming high-dimensional representational spaces (17, 20, 32, 59), potentially facilitating the integration of interdependent features. Therefore, coding interdependent features appears to involve a hybrid strategy that combines space-like and dense-like coding schemes to balance specificity and interdependency demands, significantly diverging from conventional encoding strategies for independent features.

One strength of this study lies in the two-stage methodological approach. Initially, an RNN served as a hypothesis-generating abstraction rather than a strictly biophysical model, enabling exploration of emergent solutions to the computational challenge of simultaneously encoding interdependent features. Subsequently, these hypotheses were empirically validated using neurophysiological data. Separating hypothesis generation from empirical validation allowed exploration not only of patterns in neural recordings but also of potential population structures that naturally emerge from task-driven optimization, free from predefined assumptions about neuronal tuning or connectivity. Therefore, our modeling approach was not designed to produce a precise parametric fit to empirical data; rather, it served primarily as a tool to generate testable hypotheses regarding potential neural coding solutions. Other network architectures or learning paradigms might yield comparable population structures, and it remains possible that HD systems may support multiple viable encoding strategies. Future studies are needed to explore the diversity and robustness of such coding solutions across different species, behavioral tasks, and learning histories.

Beyond the HD system, the computational challenge of simultaneously encoding interdependent features arises frequently in various sensorimotor and cognitive domains. For example, motor control of reaching movements requires the brain to coordinate hand position and velocity (52, 53). Similarly, V1 neurons (60, 61) and MT neurons jointly encode spatial position and movement velocity, exhibiting encoding properties that depend on task demands and stimulus characteristic (62). In addition, neurons in the subiculum and retrosplenial cortex exhibit task-dependent mixed selectivity, simultaneously encoding spatial position and locomotion speed (19). These examples underscore a widespread computational demand: neural circuits must preserve the specificity of individual variables while representing their dynamic coupling. Our study suggests that a hybrid population coding strategy, where mixed-selectivity neurons self-organize into functionally specialized subpopulations, offers a biologically plausible solution. This structure enhances representational geometry for dynamic features (e.g. velocity) while maintaining precise representations of stable ones (e.g. position or direction). Thus, the organizational principle revealed in the HD system may constitute a generalizable coding scheme across diverse sensorimotor and cognitive domains. Future studies are needed to systematically evaluate the prevalence and functional implications of this coding strategy within other cortical and subcortical regions that encode interdependent features.

## Materials and methods

### Recurrent neural network

The RNN for integrating AHV into HD was trained using the procedure described in Cueva et al. (23). As illustrated in Fig. 1A, the RNN contained *N* fully recurrently connected units (*N* = 100 or *N* = 64 in this study) with connection weights $W_{\;ji}^{\mathrm{rec}}$. Each unit also received three external inputs through weight matrix $W_{ki}^{\mathrm{in}}$ and projected outputs through weight matrix $W_{il}^{\mathrm{out}}$ to 2 output units. The 3 inputs were $\cos\;\theta_{t}$, $\;\sin\;\theta_{t}$ and angular velocity AHV*_t_*, which were divided into two phases:

In the first phase lasting 10 timesteps, AHV*_t_* was 0, $\cos\;\theta_{t}$ and $\sin\;\theta_{t}$ were determined by initialized HD $\theta_{0}$. In the second phase for all subsequent timesteps, $\cos\;\theta_{t}$ and $\;\sin\;\theta_{t}$ were 0, and AHV*_t_* at each timestep was simulated as $\mathrm{AH}V_{t}=\;\sigma X+\mathrm{momentum}*\mathrm{AH}V_{t-1}$ with σ=0.03 and momentum=0.8. X is a zero mean Gaussian random variable with SD of one. The seeds of the random variables changed in different simulations. The outputs were the updated $\cos\;\theta_{t}$ and $\;\sin\;\theta_{t}$ of the current step, where $\theta_{t}$ was the exact integral of all previous angular velocity. The dynamics of each recurrent unit in the RNN was updated using a biologically plausible firing rate model, which assumes that firing rate responds instantaneously to synaptic input, but synaptic conductance change slowly as shown by the following simplified input–output characteristics and differential equation for synaptic input:


$$r_{i}(t)=\;\max(0,\;\tanh(s_{i}(t)))\;$$

$$\tau_{s_{i}(t)}\frac{ds_{i}(t)}{dt}=\;-s_{i}(t)+Xi_{\mathrm{input}},\;\;i=1,2,\ldots ,\;N\;,$$

where $\tau_{s_{i}(t)}$ was set to be 250 ms for all unit and $Xi_{\mathrm{input}}$ is the summation from recurrent inputs, external inputs and constant bias:


$$Xi_{\mathrm{input}}=\;\overset{N}{\underset{\;j=1}{\sum }}\;W_{\;ji}^{\mathrm{rec}}r_{j}(t)+\overset{3}{\underset{k=1}{\sum }}\;W_{ki}^{\mathrm{in}}I_{k}(t)+b_{i}.$$

The activities of two output units were given by:


$$y_{l}(t)=\overset{N}{\underset{i=1}{\sum }}\;W_{il}^{\mathrm{out}}r_{i}(t),\;\;l=1,2.$$

The model was trained with the hessian-free algorithm under the $L_{2}$ regularization on firing rates of both recurrent and output units to minimize the mean-squared error between the target and the theoretical HD:


$$E_{l}=\sum_{t,l}{\left(y_{l}{\left(t\right)}^{\;}-y_{l}^{\mathrm{target}\;}(t)\right)}^{2}.$$

In practice, the network activities were simulated using Euler method for 500 timesteps of 25 ms duration. Parameters were optimized by minimizing mean-squared error across 500 trials in parallel to increase training efficiency. Half of the trails were allowed to have duration of AHV = 0 for up to one-third of the trial length. The model was trained for 500 epochs in total, and model parameters for epoch 500 were saved to generate dataset for further analyses.

To analyze unit deactivation effects on RNN performance, we implemented deactivations by setting the hidden states of selected units to zero after each iteration step. Performance error was quantified using normalized mean-squared error (NMSE), calculated as the percentage of squared deviation between network predictions and ground truth relative to target signal variance, and further aggregated across two outputs:


$$\begin{array}{r} \mathrm{Total}\;\mathrm{normalized}\;\mathrm{error}=\;\overset{2}{\underset{l=1}{\sum }}\;\mathrm{NMS}E_{l}\\ =\;\overset{2}{\underset{l=1}{\sum }}\;100\times \frac{E_{l}}{{\left(y_{l}^{\mathrm{target}}(t)-\mathrm{mean}(y_{l}^{\mathrm{target}})\right)}^{2}} \end{array}.$$

#### Dataset

This study analyzed datasets of firing activities from both artificial and biological neural network. The first dataset consisted of firing activity from recurrently connected units in an RNN model for specific training epoch during 200 trials with 1,000 timesteps per trial (i.e. 200,000 samples in total for each unit with a sampling interval of 25 ms). All results were derived from the firing activities generated by RNN model with parameters from training epoch 500.

The second dataset contained recorded neural spiking activity from pre-existing publicly available data “Extracellular recordings from multisite silicon probes in the anterior thalamus and subicular formation of freely moving mice” (CRCNS.org, doi:10.6080/K0G15XS1). No additional animal experiments were performed. Original data collection was approved by the Institutional Animal Care and Use Committee of New York University Medical Center as described in Peyrache et al. (45). The dataset includes recordings from five adult male and two female mice. Thirty-seven complete recording sessions across six mice were included for further analysis. For analysis of HD and AHV decoding from the neuronal populations in ADn and PoS, only sessions with >30,000 samples (ensuring small SD across cross-validation splits, Fig. S4E) and containing at least 4 neurons in both the SP and MP groups were used.

### Preprocessing of behavioral and neural data in mice

The original HD angle lists during awake states were first converted into accumulated angles from a circular distribution. The accumulated angles were then smoothed by a Gaussian kernel with *σ* = 200 ms and moving step size = 25 ms. In practice, a sliding Gaussian window G(t) of width 6 *σ* was used to increase processing speed while retaining sufficient resolution for further analyses. Angular velocity lists were obtained by convolving the accumulated angle lists with a normalized Gaussian derivative sliding window, defined as $G^{\prime }(t)=\;G(t)*(x-\mu )/\sigma^{2}$, which was subsequently normalized, for every 25 ms bin. To align the timelines between the neural firing and behavioral data, the original timestamps for spikes of each sorted neuron were convolved with the same Gaussian sliding window G(t) as above, followed by summation.

### Analysis of HD and AHV units/neurons

We utilized the same method to construct HD and AHV tuning curves for units from the RNN model and neurons from mice recordings. The 0 to 2π angular radian range was divided into 50 equal bins. For each neuron, the HD tuning curve was obtained by calculating the mean firing rate for each angular bin after averaging the instantaneous firing across all timepoints where the animal's HD direction fell within that bin. For AHV tuning curves, mean firing rates for bins of 0.2 rad/s were derived by averaging instantaneous firing for timepoints when the AHV was within each bin. Tuning curve SD were determined through 50 permutations, constructing the tuning curves each time using a randomly selected half of the full sample points. We excluded 12 units in RNN from further analysis (Fig. S2A, units with gray tuning curves) due to low tuning stability and extremely low activities (mean firing < 0.0001, Fig. S2C) throughout the simulation process.

The strength of HD tuning for each unit/neuron was assessed by computing the MI between HD and the averaged firing rates across different HD bins (MI, bits/s) defined as (28):


$$MI_{\mathrm{HD}}=\;\underset{i}{\sum }\;p(i)fr(i)\mathrm{lo}g_{2}\frac{\;fr(i)}{\;fr},\;i=1,2,\ldots ,50,$$

where fr(i) and p(i) are the mean firing rate and the probability that the animal's head pointed in the *i*th HD bin, and *fr* is the overall mean firing rate of the unit/neuron.

The strength of AHV tuning were measured as Pr_AHV_, defined as the maximum of $r_{\mathrm{AHV}\_\mathrm{pos}}$ and $r_{\mathrm{AHV}\_\mathrm{neg}}$, which represent the absolute partial Pearson's correlation coefficients (30) between firing rate and AHV for positive and negative velocities, respectively, and calculated as


$$Pr_{\mathrm{AHV}}=\mathrm{Partial}\;r(\;fr,\mathrm{ahv})=\;\frac{r(\;fr,\mathrm{ahv})-r(\;fr,\mathrm{hd})\times r(hd,\mathrm{ahv})}{\sqrt{1-r^{2}(\;fr,\mathrm{hd})}\times \sqrt{1-r^{2}(hd,\mathrm{ahv})}},$$

where *r* denotes Pearson correlation coefficients. The significance of the tuning for both HD and AHV were determined through a shuffling process. The chance-level distribution of MI_HD_ and Pr_AHV_ were derived from 1,000 repetitions with shuffled condition. For each repetition, the firing rate time series was shifted by a random interval of at least 30 s, with the end of the trial wrapped to the beginning. Significantly tuned HD and AHV units and neurons were defined when calculated MI_HD_ and Pr_AHV_ values were higher than 99th percentile of the shuffled distribution.

### Classification of SP and MP HD units/neurons

In the RNN model, SP and MP HD units were separated by manual inspection. MP units dominated population 1 (11 of 13 units, Fig. 1C) and showed the lowest MI_HD_ in the network. In mouse recordings, SP and MP HD neurons were classified using a two-step method. A HD-tuned neuron was labeled as SP if: (i) no bins in its HD tuning curve (outside the half-peak range) had a mean firing rate exceeding half of the maximum response. (ii) Fewer than *α* of bins (outside the 20% peak range) had firing rates above 20% of the maximum response. Three *α* thresholds were tested (20, 10, and 5%), with lower values making the SP classification stricter. In order to quantify the MP nature of the tuning, we calculated MVL for each unit/neuron, which typically was used to describe the strength of HD tuning for conventional SP neurons. MVL was defined as following:


$$\left|\frac{\sum \lambda_{i}e^{\;j\theta_{i}}}{\sum \lambda_{i}}\right|,$$

where *j* is the imaginary unit, $\theta_{i}$ is the HD angle in radians associated with the *i*th bin, and $\lambda_{i}$ is the mean firing rate in that bin. By applying MVL, a metric normally used for SP tuning, to MP neurons, we could quantify the degree of multiphasic in their tuning curves.

### Feedforward neural network

We assessed the encoding capability of neuronal populations using a 5-fold cross-validation with well-trained artificial neural networks (ANNs). The underlying hypothesis was that a feedforward ANN, known as a universal approximator, could extract the maximal information from the population activity patterns.

Two ANNs were trained for each population group (SP or MP) in both the RNN and mice datasets—one for predicting HD and one for predicting AHV. Both ANNs had three fully connected hidden layers with 64, 128, and 64 units respectively. Each unit in a layer was computed from a linear combination of the previous layer's outputs passed through a rectified linear activation function called ReLU (63). The Adam optimization algorithm was used (64), with weight decay set to 5e−2 and 5e−3 for the HD and AHV ANNs, respectively.

The input to the ANNs was the instantaneous firing rates of the neuronal populations. The output was either the corresponding HD or AHV value at each timepoint. For HD predictions, we applied a modulo 2π operation to constrain all angles within the [0, 2π] range, ensuring proper representation on the circular domain (see Fig. 3A). To quantify decoding performance, we computed Pearson correlation coefficients (decoding *r*) between the target and predicted values. For HD correlations, both predicted and target variables were cosine-transformed prior to analysis to account for circularity.

### Measurement of FWHM for SP tuning and MP tuning curves

We calculated the FWHM of neuronal tuning curves using the following method: First, we normalized each tuning curve by subtracting its minimum value. For SP neurons, we identified the peak firing rate and then determined the angular positions (HD_HM_left and HD_HM_right) where the firing rate dropped to half of this peak value on either side—the angular difference between these points gave the FWHM. For MP neurons, we first detected all local peaks (set distance parameters as 10 for RNN units and 8 for recorded neurons when using find_peaks function), then for each qualified peak (with at least one flank dropping below half-maximum), we similarly measured either the full angular span between half-max points (if both sides were present) or twice the half-span (if only one side was detectable). The final FWHM for MP neurons was calculated as the mean of all qualified peak widths, with averages of 2.2 peaks per RNN unit, 3.5 for ADn neurons, and 3.3 for PoS neurons. It's worthy to note that for SP units, the FWHM was measured around the single global peak of the tuning curve. For MP units, we first detected all local peaks using a minimum separation threshold and then calculated the FWHM for each qualified peak individually. The reported FWHM for MP units is the mean of these individual peak widths, rather than the total angular spread across all peaks. This allows for a meaningful comparison of local tuning sharpness, which contributes to manifold complexity and AHV representation (32), while avoiding inflation due to peak multiplicity or inter-peak gaps.

### Effective dimensionality

According to Ref. (51), the ED of a set of S points $\;V=[V_{sk}]\in \;R^{SXd}$ with embedding dimension d is


$$\mathrm{ED}(V)=\;\frac{\mathrm{Tr}{\left(C\right)}^{2}}{\mathrm{Tr}(C^{2})}=\;\frac{{\left(\sum_{k=1}^{d}\;\lambda_{k}\right)}^{2}}{\left(\sum_{k=1}^{d}\;\lambda_{k}^{2}\right)}={\left(\overset{d}{\underset{k=1}{\sum }}\;{\tilde{\lambda_{k}}}^{2}\right)}^{-1},$$

where Tr is the trace operation for matrices, *C* is the covariance matrix of *V*, and $\tilde{\lambda_{k}}=\lambda_{k}/\underset{j}{\sum }\;\lambda_{j}$ are the normalized eigenvalues of *C*. Therefore, to calculate ED for each subpopulation of 11 units analyzed in the RNN model, the first step was to construct an activity matrix *T* of size 11*200,000, where each row represented the activity time series of a unit in that group during a simulation session. Then, PCA analysis was performed on the transpose of the *T* to obtain explained variance ratio of each PCA component, i.e. $\tilde{\lambda_{k}}$. Thus, the ED could be calculated according to the above equation.

### Characterization of the HD manifold in high-dimensional activity space of RNN

To isolate how response profiles shape population geometry while eliminating metabolic cost artifacts from RNN training, we z-scored each unit's firing rates before characterizing the head-direction (HD) manifold by binning 0–2π into 360 angular segments and averaging firing rates to construct an 11-dimensional representation, with trajectory length (${L\_}_{\mathrm{trajectory}}$) calculated as cumulative Euclidean distances between adjacent bin states; for visualization, we projected the manifold onto the first three principal components (Fig. 5D and E) and normalized down-sampled states by maximum PC values. To assess MP unit contributions, we systematically replaced 2–9 SP units (20–80% replacement) with MP units across 7 SP subpopulations and quantified changes in manifold richness through trajectory length differences (${\Delta L\_}_{\mathrm{trajectory}}$) and decoding *r* (HD/AHV) compared to pure SP groups, with results averaged over 20 replicates (Fig. 5F and G).

### Software and statistical analysis

The RNN model training and data generation were performed in MATLAB 2021a (Mathworks), using code generously shared by Cueva et al. (23). Subsequent analyses and statistical test of the simulated RNN unit activities and recorded neuronal firing were all conducted in Python using the VScode environment.

## Supplementary Material

pgaf320_Supplementary_Data

## Data Availability

RNN simulation data and analysis code are accessible through GitHub repository: https://github.com/dongqin/HD-AHV-ANN-decoding-analysis. All analyses used custom Python (3.9) and MATLAB (2021a) code. The mouse data supporting this study are available at CRCNS.org (https://portal.nersc.gov/project/crcns/download/th-1).

## Preprints

This manuscript was posted on a preprint: https://www.biorxiv.org/content/10.1101/2024.05.10.593505v1.
