# Supplementary material for: Encoding of interdependent features of head direction and angular head velocity in navigation
Source: PNAS Nexus. 2025 Oct 9;4(10):pgaf320. doi: 10.1093/pnasnexus/pgaf320 (PMC12550893; doi:10.1093/pnasnexus/pgaf320)
Supplement: pgaf320_Supplementary_Data [file pgaf320_supplementary_data.pdf]

PNAS nexus

Supplementary Information for

Encoding of interdependent features of head direction and angular head velocity in navigation

Dongqin Cai, Tao Liu, Jia Liu*
Tsinghua Laboratory of Brain and Intelligence, Department of Psychological and Cognitive Sciences, Tsinghua University, Beijing, China
* To whom correspondence should be addressed to: Email: liujiathu@tsinghua.edu.cn (Jia Liu)

This pdf file includes:
Supplementary Figures 1 to 5 and their corresponding legends.

Supplementary Figure 1

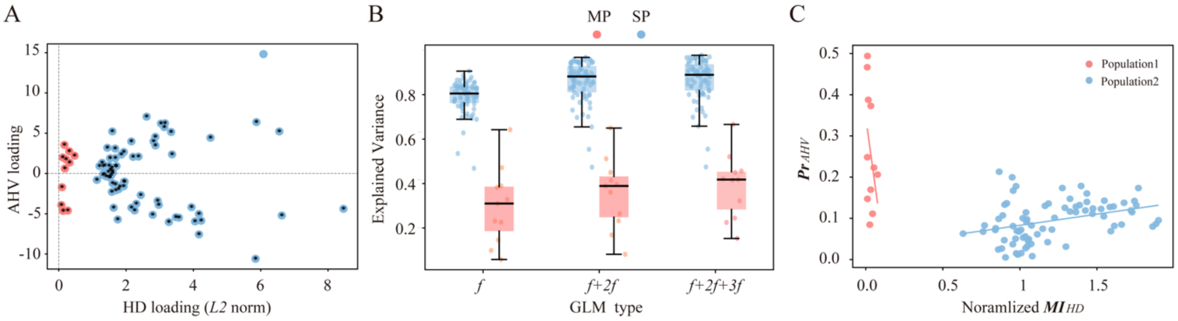

Fig. S1. GLM analysis distinguishing encoding preferences for SP and MP units. (A) Scatter plot comparing AHV loadings versus HD loadings (quantified as the L2 norm of coefficients derived from sine and cosine predictors) for SP and MP units. *: units with significant loadings for both AHV and HD. All units except one SP unit showed significant loadings. (B) Scatter and box plots illustrating explained variance (showing 1st and 3rd quartiles) for SP and MP units across different GLM configurations: fundamental frequency sine/cosine basis (*f*), combined fundamental and first harmonic bases (*f* + *2f*), or fundamental, first, and second harmonic bases (*f* + *2f* + *3f*). SP units consistently showed significantly higher explained variance than MP units across all model configurations ($p < 0.001$, Mann–Whitney U test). (C) Scatter plot illustrating the relationship between MI_{HD} (bits/spike) and AHV encoding strength (Pr_{AHV}), indicating robustness of the distinction between population1 (consisting exclusively of MP units) and population2 (consisting exclusively of SP units) using mutual information (MI_{HD}) normalized by mean firing rate (bit/spike). Blue regression line indicates SP units (Population 2; slope $\rho = 0.05$, $p < 0.05$); red regression

line indicates MP units (Population 1; slope ρ= -2.72, p = 0.2). The clear separation between MP and SP units remains consistent with our original results obtained using MI in bits/sec.

Supplementary Figure 2

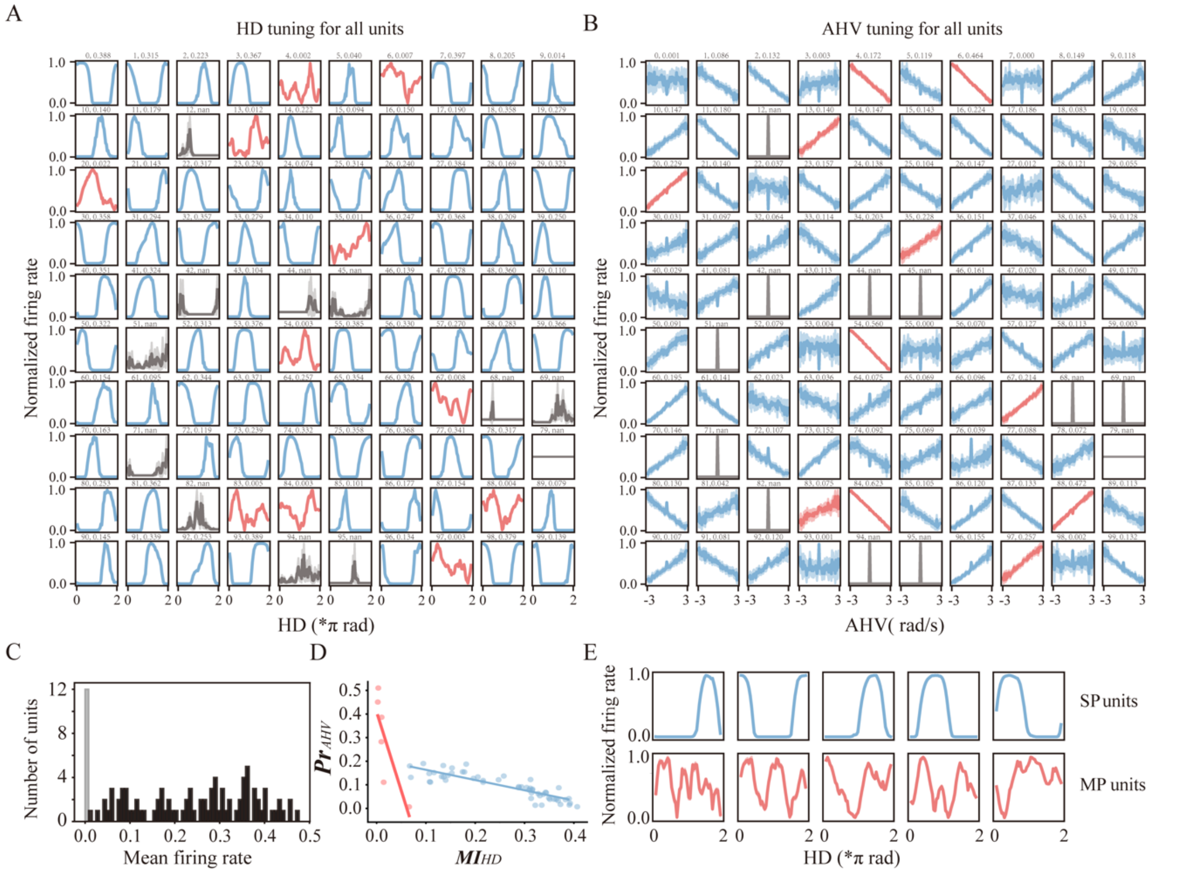

Fig. S2. Emergence of SP and MP units of HD in trained RNNs with network size of both 100 and 64, related to Fig. 2. (A and B) Tuning curves of HD and AHV for all units in 100-unit RNN. Units with gray tuning curves are excluded from further analysis due to low mean firing rate and unstable tuning. SP units are represented in blue, while MP units are denoted in red. This color code for SP and MP units remains consistent throughout all subsequent figures. Shaded areas represent the standard deviation of tuning curves obtained from 50 permutations of the original firing and HD sequences. Shaded areas are not visible for SP and MP units due to negligible standard deviation across permutations. Unit number and MI_{HD} are labeled at the top of each HD tuning plot in (A); unit number and Pr_{AHV} are labeled at the top of each AHV tuning plot in (B). In (B), only the tuning curves with AHV values in the range of [-3,3] are shown for a better comparison across units. MI_{HD} and Pr_{AHV} values of nan represent units excluded for analysis due to excessively low mean firing rates observed throughout the simulation. (C) Distribution of mean firing rates for all units, gray units in (A and B) are included in the histogram around 0. (D and E) Emergence of two populations in the 64-unit RNN. (D) Relationship between the representations of HD and AHV for 59 units significantly tuned to HD in the 64-unit RNN. Blue linear regression

line, ρ= -2.995, p = 0.064; red linear regression line, ρ=-0.254, p = 1.12*e-13. (E) Tuning curves of HD for five units with the highest MI_{HD} (upper row) and five units with the lowest MI_{HD} (bottom row) in the 64-unit RNN.

Supplementary Figure 3

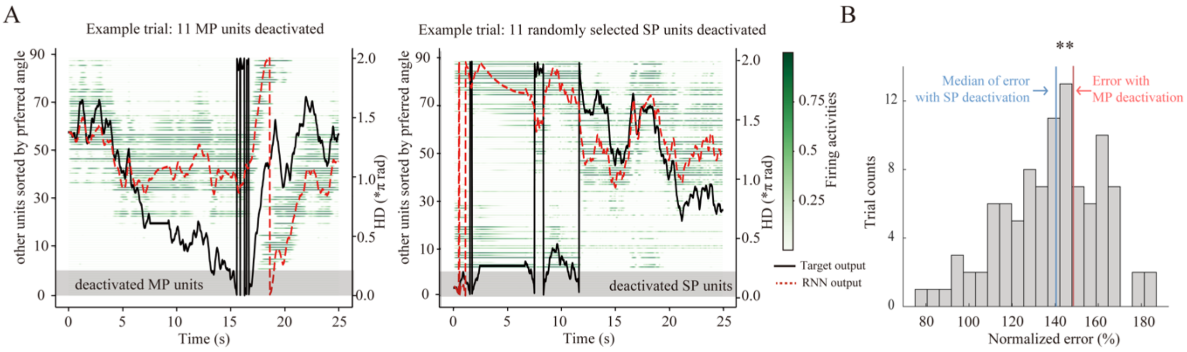

Fig. S3. Loss function experiments reveal the critical role of MP units in network integration of AHV into HD representation. (A, B) Superimposed traces show predicted HD (red), ground truth HD (black), and population firing rates (green, with intensity indicating normalized rates) from an example test trial after deactivating either 11 MP units (A) or 11 randomly selected SP units (B), with y-axis showing unit identity. (C) Distribution of normalized HD errors across 100 trials of SP unit deactivation (11 units/trial) demonstrates significantly lower

median error for SP deactivation compared to MP deactivation (Wilcoxon rank-sum test, $p < 0.01$), indicating MP units' greater importance for network performance when equal numbers of units are deactivated.

Supplementary Figure 4

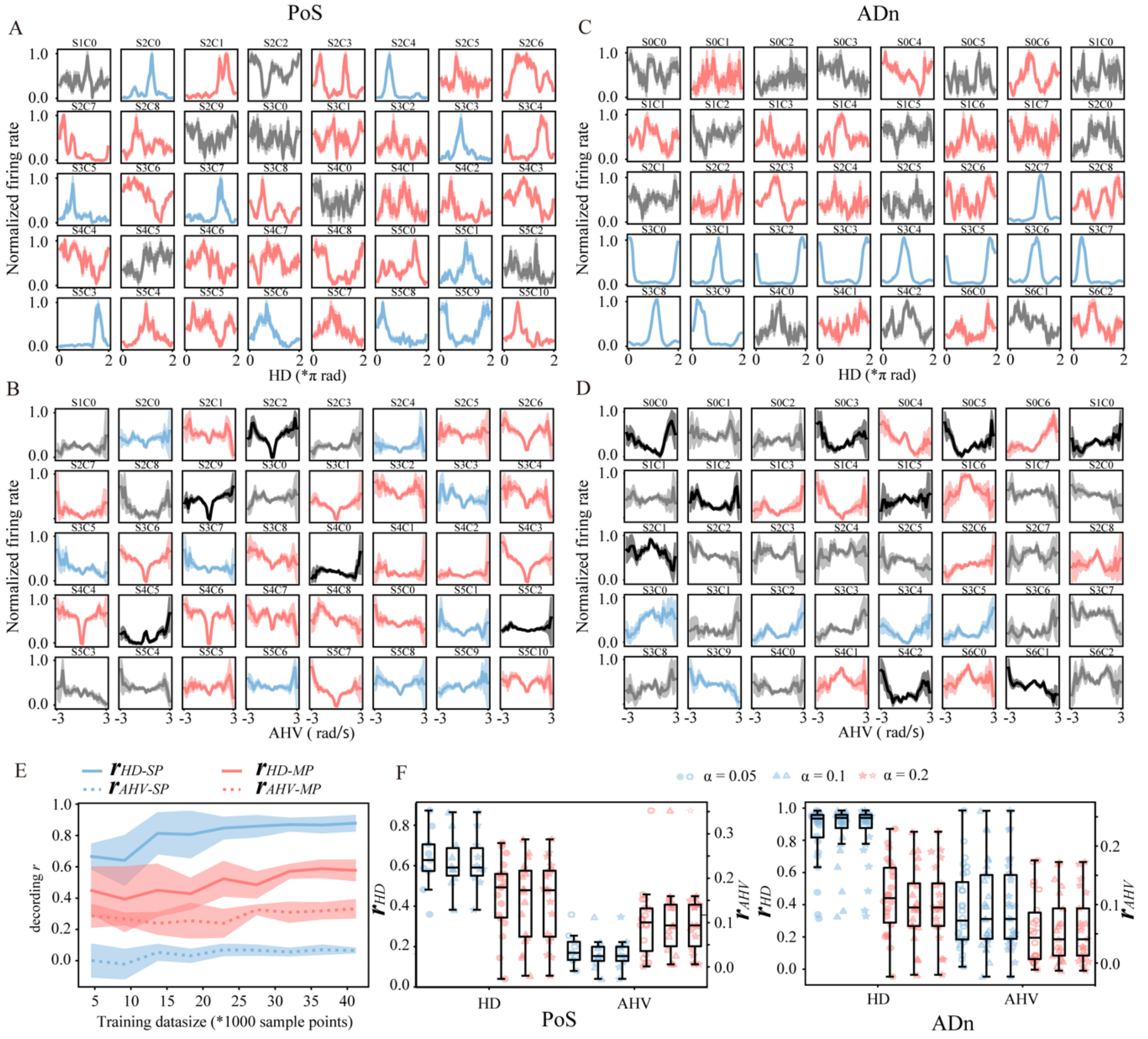

Fig. S4. Detailed examples and additional analysis, related to Fig. 4. (A and B) HD and AHV tuning curves for neurons recorded in PoS during exemplar session Mouse28-140312. Blue and red neurons in (A) are HD-tuned and classified as SP and MP, respectively. In (B), black, blue, and red neurons are AHV-tuned; among them, only blue and red neurons are also significantly HD-tuned, representing SP and MP HD neurons, respectively. Shaded areas represent standard deviation of tuning curves obtained from 50 permutations of the original firing rates, HD and AHV sequences. Unit shank and cluster are labeled at the top of each tuning plot. (C and D) same as (A and B) but for neurons in ADn during exemplar session Mouse12-120808. (E) Change in decoding correlations when increasing training data size for the illustrated session in (A). To ensure robust estimates with minimal variability across validation splits, we restricted analysis to sessions containing >30,000 samples. The solid and dashed lines represent the mean decoding correlations for HD and AHV, respectively. Shaded areas represent the standard deviation of all five-fold leave-one-out tests. (F) Scatter and box plots comparing decoding accuracy (r) for HD and AHV between SP and MP neuron populations across all recording sessions from PoS (left) and ADn (right), analyzed using three distinct strictness parameters (α). In PoS, three-way ANOVA revealed significant main effects of population type (SP vs MP: $F(1,154)=7.355$, $p<0.001$) and variable type (HD vs AHV: $F(1,154)=394.72$, $p<0.001$), with a significant interaction ($F(1,154)=33.86$, $p<0.001$). Similarly in ADn, we found significant effects of population type ($F(1,284)=167.67$, $p<0.001$) and variable type ($F(1,284)=1065.14$, $p<0.001$),as well as their interaction ($F(1,284)=120.41$, $p<0.001$). The strictness parameter α showed no significant effect in either region

(PoS: F(2,153) = 0.12, p = 0.89; ADn:F(2,283) = 0.035, p = 0.97). Statistical significance markers for each group indicate differences from 0 based on Wilcoxon signed-rank tests: *p<0.05,**p<0.01, ***p<0.001.

Supplementary Figure 5

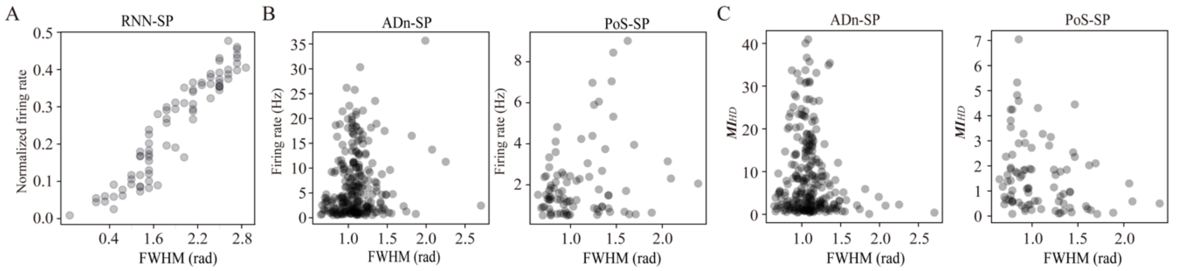

Fig. S5. Firing rate and mutual information as a function of FWHM. (A) Firing rate increases with FWHM in RNN units (r = 0.94, p < 0.001). (B) Firing rate increases with FWHM in ADn (r = 0.12, p = 0.04) and PoS neurons (r = 0.25, p = 0.017). (C) There is a positive linear correlation between MI_{HD} and FWHM for 79% ADn neurons (for FWHM < 1.23 rad, r = 0.17, p = 0.01) and 31% PoS neurons (for FWHM < 0.85 rad, r = 0.4, p = 0.03).

Supplementary Figure 6

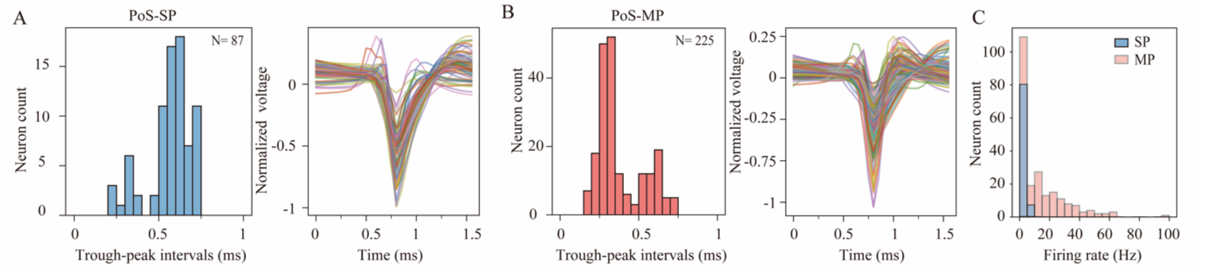

Fig. S6. Distinct electrophysiological signatures of SP and MP neurons in PoS. (A) Distribution of trough-to-peak intervals and superimposed waveforms for SP neurons. (B) Corresponding distributions and waveforms for MP neurons. (C) Distribution of mean firing rate of SP and MP neurons.
